# Supplementary material for: The mother’s risk of premature death after child loss across two centuries
Source: eLife. 2019 Nov 12;8:e43476. doi: 10.7554/eLife.43476 (PMC6850766; doi:10.7554/eLife.43476)
Supplement: Supplementary file 1. [file elife-43476-supp1.docx]

**Supplementary Table 1. Descriptive characteristics of parents born from 1800 to 1996 who lost a child by birth cohorts in the population-based matched cohort, N (%).**

|  | **Birth cohort 1800-1996** | | **Birth cohort 1800-1880** | | **Birth cohort 1881-1930** | | **Birth cohort 1931-1996** | |
| --- | --- | --- | --- | --- | --- | --- | --- | --- |
|  | Parents with loss | Parents without loss | Parents with loss | Parents without loss | Parents with loss | Parents without loss | Parents with loss | Parents without loss |
| Total number | 64,044 | 218,824 | 35,441 | 106,302 | 19,281 | 65,913 | 9,322 | 46,609 |
| % of the exposed in population | 20.6 | - | 61.1 | - | 26.9 | - | 5.2 | - |
| Sex |  |  |  |  |  |  |  |  |
| Female | 30,539 (48) | 30,539 (47) | 17,353 (49) | 17,353 (49) | 8,984 (47) | 8,984 (46) | 4,202 (45) | 4,202 (45) |
| Male | 33,505 (52) | 33,505 (53) | 18,088 (51) | 18,088 (51) | 10,297 (53) | 10,297 (54) | 5,120 (55) | 5,120 (55) |
| Total number of children | 368,875 | 942,611 | 226,871 | 528,814 | 104,797 | 266,028 | 37,207 | 147,769 |
| Number of children, mean(SD) | 5.76 (3.20) | 4.31 (2.78) | 6.40 (3.50) | 4.97 (3.21) | 5.44 (2.83) | 4.04 (2.44) | 3.99 (1.57) | 3.17 (1.38) |
| Number of children |  |  |  |  |  |  |  |  |
| 1 | 2,638 (4) | 2,638 (12) | 1,630 (5) | 1,630 (12) | 746 (4) | 746 (12) | 262 (3) | 262 (9) |
| 2-4 | 23,916 (37) | 23,916 (52) | 10,238 (29) | 10,238 (39) | 7,495 (39) | 7,495 (54) | 6,183 (66) | 6,183 (77) |
| 5-9 | 29,241 (46) | 29,241 (31) | 17,117 (48) | 17,117 (39) | 9,302 (48) | 9,302 (31) | 2,822 (30) | 2,822 (14) |
| 10+ | 8,249 (13) | 8,249 (6) | 6,456 (18) | 6,456 (9) | 1,738 (9) | 1,738 (3) | 55 (1) | 55 (0) |
| Age at first child birth, mean(SD) | 25.89 (5.27) | 25.63 (5.31) | 27.17 (5.25) | 26.70 (5.20) | 25.27 (4.86) | 25.91 (5.36) | 22.31 (4.16) | 22.81 (4.40) |
| Age at loss or match, mean(SD) | 40.39 (16.53) | 40.99 (16.92) | 36.98 (13.33) | 36.99 (13.28) | 45.82 (19.57) | 46.60 (19.89) | 42.12 (17.37) | 42.16 (17.33) |
| Age at loss or match |  |  |  |  |  |  |  |  |
| 13-30 | 21,968 (34) | 21,968 (34) | 13,523 (38) | 13,523 (38) | 5,294 (27) | 5,294 (27) | 3,151 (34) | 3,151 (33) |
| 31-50 | 26,997 (42) | 26,997 (41) | 16,499 (47) | 16,499 (47) | 7,226 (37) | 7,226 (36) | 3,272 (35) | 3,272 (35) |
| 51-75 | 11,842 (18) | 11,842 (20) | 4,802 (14) | 4,802 (14) | 4,561 (24) | 4,561 (24) | 2,479 (27) | 2,479 (27) |
| 76+ | 3,237 (5) | 3,237 (6) | 617 (2) | 617 (2) | 2,200 (11) | 2,200 (12) | 420 (5) | 420 (4) |
| Age of deceased child, mean(SD) | 10.62 (16.16) | - | 6.04 (11.55) | - | 16.40 (19.59) | - | 16.11 (17.72) | - |
| Length of follow-up, mean(SD) | 30.12 (18.45) | 23.48 (17.60) | 30.57 (17.73) | 20.68 (16.59) | 32.26 (19.94) | 28.00 (19.06) | 24.03 (16.55) | 23.47 (16.32) |
| Age at death | 70.72 (16.62) | 71.67 (16.23) | 67.53 (16.76) | 67.37 (16.86) | 77.30 (14.64) | 77.55 (13.97) | 67.11 (12.50) | 67.60 (12.21) |

**Supplementary Table 2. Descriptive characteristics of female and male parents born from 1800 to 1996 who lost a child by death during their life course, and their sisters and brothers who did not lose a child, N (%).**

|  | **Female parents with loss** | **Sisters without loss** | **Brothers without loss** | **Male parents with loss** | **Brothers without loss** | **Sisters without loss** |
| --- | --- | --- | --- | --- | --- | --- |
| Total number | 25,125 | 34,018 | 33,256 | 22,586 | 29,758 | 29,310 |
| Total number of children | 141,889 | 139,807 | 145,967 | 134,930 | 128,780 | 117,182 |
| Number of children, mean(SD) | 5.65 (3.07) | 4.11 (2.58) | 4.39 (2.89) | 5.97 (3.31) | 4.33 (2.83) | 4.00 (2.51) |
| Number of children |  |  |  |  |  |  |
| 1 | 1,057 (4.21) | 4,509 (13.25) | 3,921 (11.79) | 718 (3.18) | 3,526 (11.85) | 4,056 (13.84) |
| 2-4 | 9,575 (38.11) | 17,558 (51.61) | 16,744 (50.35) | 8,191 (36.27) | 15,194 (51.06) | 15,412 (52.58) |
| 5-9 | 11,504 (45.79) | 10,485 (30.82) | 10,468 (31.48) | 10,443 (46.24) | 9,300 (31.25) | 8,766 (29.91) |
| 10+ | 2,989 (11.90) | 1,466 (4.31) | 2,123 (6.38) | 3,234 (14.32) | 1,738 (5.84) | 1,076 (3.67) |
| Age at first child birth, mean(SD) | 24.35 (4.70) | 25.54 (5.40) | 28.57 (6.33) | 26.89 (5.17) | 28.67 (6.42) | 25.92 (5.53) |
| Age at matching^*^, mean(SD) | 38.04 (15.83) | 36.87 (15.32) | 36.10 (14.49) | 39.56 (14.85) | 37.95 (13.80) | 38.69 (14.51) |
| Age at matching^*^ |  |  |  |  |  |  |
| 13-30 | 10,490 (41.75) | 15,460 (45.45) | 15,398 (46.30) | 7,473 (33.09) | 11,022 (37.04) | 10,554 (36.01) |
| 31-50 | 9,602 (38.22) | 12,439 (36.57) | 12,397 (37.28) | 10,378 (45.95) | 13,461 (45.23) | 13,051 (44.53) |
| 51-75 | 4,103 (16.33) | 5,141 (15.11) | 4,727 (14.21) | 4,020 (17.80) | 4,709 (15.82) | 4,903 (16.73) |
| 76+ | 930 (3.70) | 978 (2.87) | 734 (2.21) | 715 (3.17) | 566 (1.90) | 802 (2.74) |
| Age of deceased child, mean(SD) | 9.99 (15.59) | - | - | 8.82 (14.18) | - | - |
| Length of follow-up, mean(SD) | 33.64 (18.94) | 27.10 (18.96) | 24.17 (17.55) | 29.73 (17.47) | 23.85 (17.02) | 26.62 (18.22) |
| Age at death | 72.03 (17.06) | 73.78 (16.10) | 69.46 (16.36) | 69.51 (16.12) | 70.11 (15.98) | 74.01 (15.77) |

^*^ Age at loss of first child for parents who lost a child, as well as the same age for their siblings who did not lose a child or the age when the siblings became parents, whichever came later.

**Supplementary Table 3. Hazard ratios (HRs) and 95% confidence intervals (CIs) of premature mortality after loss of a child among young parents who were followed from age 51 onward, by time since loss and demographic characteristics, compared to their siblings.** We stratified on sibling groups and additionally adjusted for birth year and sex. IR, incidence rate, per 1000 person-years.

|  | **Overall** | | **Women** | | **Men** | |
| --- | --- | --- | --- | --- | --- | --- |
|  | **N (Crude IR)** | **HR (95% CI)** | **N (Crude IR)** | **HR (95% CI)** | **N (Crude IR)** | **HR (95% CI)** |
| **Time since loss** |  |  |  |  |  |  |
| 0-4 years^*^ | 1,491 (30.7) | 0.95 (0.88-1.01) | 639 (25.5) | 0.91 (0.81-1.03) | 852 (36.1) | 1.01 (0.90-1.14) |
| 5-9 years | 1,258 (26.7) | 1.08 (1.00-1.16) | 534 (22.3) | 0.98 (0.86-1.12) | 724 (31.2) | 1.15 (1.01-1.30) |
| 10-19 years | 2,875 (24.1) | 0.98 (0.93-1.03) | 1,291 (21.4) | 1.03 (0.94-1.13) | 1,584 (26.9) | 0.97 (0.89-1.05) |
| 20-39 years | 9,086 (26.8) | 1.00 (0.97-1.03) | 4,312 (23.2) | 1.01 (0.96-1.07) | 4,774 (31.0) | 0.99 (0.94-1.04) |
| **Child’ age at loss** |  |  |  |  |  |  |
| 0 | 10,670 (36.9) | 1.02 (0.99-1.06) | 5,463 (35.0) | 1.07 (1.01-1.13) | 5,207 (39.2) | 0.98 (0.93-1.04) |
| 1-5 | 7,552 (37.6) | 0.98 (0.94-1.02) | 3,856 (35.3) | 1.01 (0.94-1.08) | 3,696 (40.2) | 0.97 (0.91-1.04) |
| 6-17 | 2,843 (34.8) | 1.03 (0.97-1.09) | 1,447 (32.3) | 1.03 (0.93-1.14) | 1,396 (37.8) | 1.06 (0.95-1.17) |
| 18+ | 5,353 (38.3) | 1.04 (0.99-1.09) | 2,772 (34.3) | 0.98 (0.91-1.05) | 2,581 (43.8) | 1.08 (1.00-1.16) |
| **Number of alive children at loss** |  |  |  |  |  |  |
| 0 | 6,688 (38.7) | 1.01 (0.97-1.05) | 3,583 (36.7) | 1.01 (0.95-1.09) | 3,105 (41.2) | 0.99 (0.92-1.06) |
| 1-3 | 14,935 (37.5) | 1.00 (0.98-1.03) | 7,636 (35.0) | 1.03 (0.98-1.07) | 7,299 (40.5) | 1.01 (0.97-1.06) |
| 4+ | 7,515 (41.9) | 1.03 (0.99-1.07) | 3,834 (38.3) | 1.01 (0.95-1.08) | 3,681 (46.5) | 1.04 (0.98-1.11) |
| **Sex of the lost child** |  |  |  |  |  |  |
| Female | 12,334 (39.5) | 1.00 (0.97-1.03) | 6,376 (37.0) | 1.01 (0.96-1.06) | 5,958 (42.6) | 0.99 (0.94-1.05) |
| Male | 16,804 (38.3) | 1.02 (0.99-1.05) | 8,677 (35.7) | 1.03 (0.99-1.07) | 8,127 (41.6) | 1.03 (0.98-1.07) |
| **Age at loss** |  |  |  |  |  |  |
| 13-30 | 8,767 (35.3) | 1.04 (1.01-1.08) | 5,233 (33.8) | 1.07 (1.01-1.13) | 3,534 (37.6) | 1.01 (0.94-1.08) |
| 31-40 | 8,643 (37.4) | 0.98 (0.94-1.02) | 4,216 (35.4) | 1.02 (0.96-1.09) | 4,427 (39.5) | 0.97 (0.91-1.03) |
| 41-50 | 4,370 (34.6) | 1.00 (0.95-1.05) | 2,012 (31.6) | 1.01 (0.93-1.10) | 2,358 (37.8) | 1.02 (0.94-1.10) |
| 51+ | 7,358 (50.8) | 1.02 (0.98-1.06) | 3,592 (46.0) | 0.96 (0.90-1.03) | 3,766 (56.4) | 1.07 (1.01-1.14) |
| **Age at first childbirth** |  |  |  |  |  |  |
| 13-21 | 5,089 (31.1) | 1.08 (1.03-1.13) | 3,799 (30.5) | 1.06 (1.00-1.13) | 1,290 (33.1) | 1.16 (1.05-1.29) |
| 22-24 | 6,833 (37.3) | 1.02 (0.98-1.07) | 4,066 (36.5) | 1.04 (0.97-1.10) | 2,767 (38.7) | 1.02 (0.95-1.09) |
| 25-27 | 6,734 (40.5) | 0.98 (0.94-1.02) | 3,206 (38.8) | 0.98 (0.91-1.05) | 3,528 (42.2) | 1.00 (0.94-1.07) |
| 28+ | 10,482 (44.1) | 0.99 (0.95-1.02) | 3,982 (41.0) | 1.00 (0.93-1.06) | 6,500 (46.1) | 0.99 (0.94-1.04) |

^*^ No parents died on the same day with the child.

**Supplementary Table 4. Hazard ratios (HRs) and 95% confidence intervals (CIs) of parental mortality after loss of a child among parents born during 1931-1996 and followed from age 51 onward, by number of children by age 50 and sex of the deceased child, in the sibling cohort.** We stratified by sibling groups and additionally adjusted for birth year and sex in Cox proportional hazards model.

| **N of Children by age 50** | **Sex of deceased child** | **Overall** | | **Women** | | **Men** | |
| --- | --- | --- | --- | --- | --- | --- | --- |
|  |  | **N (Crude IR)** | **HR (95% CI)** | **N (Crude IR)** | **HR (95% CI)** | **N (Crude IR)** | **HR (95% CI)** |
| 0 | Any | 43 (16.3) | 3.11 (1.89-5.11) | 24 (14.0) | 2.74 (1.30-5.77) | 19 (20.8) | 6.69 (2.18-20.53) |
|  | Daughter | 17 (17.9) | 2.72 (1.26-5.89) | 7 (12.1) | 2.84 (0.78-10.38) | 10 (26.8) | 4.39 (1.26-15.27) |
|  | Son | 26 (15.5) | 3.40 (1.78-6.48) | 17 (14.9) | 2.71 (1.19-6.15) | 9 (16.6) | 17.74 (2.82-111.65) |
| 1-3 | Any | 783 (11.6) | 1.16 (1.04-1.28) | 363 (10.0) | 1.17 (0.99-1.38) | 420 (13.5) | 1.22 (1.04-1.43) |
|  | Daughter | 283 (11.8) | 1.20 (1.01-1.42) | 126 (10.1) | 1.31 (1.00-1.70) | 157 (13.8) | 1.18 (0.93-1.51) |
|  | Son | 500 (11.5) | 1.13 (1.00-1.29) | 237 (10.0) | 1.11 (0.91-1.35) | 263 (13.3) | 1.24 (1.03-1.50) |
| 4+ | Any | 593 (15.5) | 1.01 (0.90-1.13) | 289 (13.4) | 0.99 (0.83-1.18) | 304 (18.4) | 1.13 (0.93-1.36) |
|  | Daughter | 213 (15.3) | 1.15 (0.95-1.38) | 110 (13.5) | 1.10 (0.85-1.44) | 103 (18.0) | 1.33 (0.98-1.79) |
|  | Son | 380 (15.7) | 0.94 (0.82-1.09) | 179 (13.3) | 0.93 (0.75-1.15) | 201 (18.6) | 1.04 (0.83-1.30) |
