# Supplementary material for: The mother’s risk of premature death after child loss across two centuries
Source: eLife. 2019 Nov 12;8:e43476. doi: 10.7554/eLife.43476 (PMC6850766; doi:10.7554/eLife.43476)
Supplement: Transparent reporting form [file elife-43476-transrepform.docx]

***eLife’s* transparent reporting form**

We encourage authors to provide detailed information *within their submission* to facilitate the interpretation and replication of experiments. Authors can upload supporting documentation to indicate the use of appropriate reporting guidelines for health-related research (see [EQUATOR Network](http://www.equator-network.org/%20)), life science research (see the [BioSharing Information Resource](https://biosharing.org/)), or the [ARRIVE guidelines](http://www.plosbiology.org/article/info:doi/10.1371/journal.pbio.1000412) for reporting work involving animal research. Where applicable, authors should refer to any relevant reporting standards documents in this form.

If you have any questions, please consult our Journal Policies and/or contact us: [editorial@elifesciences.org](mailto:editorial@elifesciences.org).

**Sample-size estimation**

- You should state whether an appropriate sample size was computed when the study was being designed
- You should state the statistical method of sample size computation and any required assumptions
- If no explicit power analysis was used, you should describe how you decided what sample (replicate) size (number) to use

Please outline where this information can be found within the submission (e.g., sections or figure legends), or explain why this information doesn’t apply to your submission:

We included all parents from the total population during the study period, 1817-2015, or 282,868 parents of which 64,044 lost at least one child. A previous Danish study of total mortality among bereaved parents in contemporary times including roughly 20,000 exposed parents and found significant associations between child loss and total parent mortality rates. We therefore concluded that we had sufficient power for the planned analyses.

**Replicates**

- You should report how often each experiment was performed
- You should include a definition of biological versus technical replication
- The data obtained should be provided and sufficient information should be provided to indicate the number of independent biological and/or technical replicates
- If you encountered any outliers, you should describe how these were handled
- Criteria for exclusion/inclusion of data should be clearly stated
- High-throughput sequence data should be uploaded before submission, with a private link for reviewers provided (these are available from both GEO and ArrayExpress)

Please outline where this information can be found within the submission (e.g., sections or figure legends), or explain why this information doesn’t apply to your submission:

Given the 200 years study period, it is indeed challenging to find a replication setting. However, we have performed analyses by birth cohorts whenever possible and our findings on increased mortality rate among bereaved parents in contemporary times resemble findings of previous investigators (Li et al., Lancet, 2003). Also, the consistent pattern of elevated maternal mortality rates across birth cohorts (for example, Figures 1 and 2), also in pre-industrial times, is supported by reports of intense emotional reactions of mothers to child loss in contemporary developing countries.

**Statistical reporting**

- Statistical analysis methods should be described and justified
- Raw data should be presented in figures whenever informative to do so (typically when N per group is less than 10)
- For each experiment, you should identify the statistical tests used, exact values of N, definitions of center, methods of multiple test correction, and dispersion and precision measures (e.g., mean, median, SD, SEM, confidence intervals; and, for the major substantive results, a measure of effect size (e.g., Pearson's r, Cohen's d)
- Report exact p-values wherever possible alongside the summary statistics and 95% confidence intervals. These should be reported for all key questions and not only when the p-value is less than 0.05.

Please outline where this information can be found within the submission (e.g., sections or figure legends), or explain why this information doesn’t apply to your submission:

The statistical analysis has been thoroughly described in the Methods (pages 12-14). We have presented N and incidence rates in all tables/figures. P values for HRs are not reported given confidence intervals are already informative on statistical significance. However, we are certainly willing to reconsider our position on this issue on the editors’ request. Of note, we did report P for difference when comparing HRs from different birth cohorts.

(For large datasets, or papers with a very large number of statistical tests, you may upload a single table file with tests, Ns, etc., with reference to sections in the manuscript.)

**Group allocation**

- Indicate how samples were allocated into experimental groups (in the case of clinical studies, please specify allocation to treatment method); if randomization was used, please also state if restricted randomization was applied
- Indicate if masking was used during group allocation, data collection and/or data analysis

Please outline where this information can be found within the submission (e.g., sections or figure legends), or explain why this information doesn’t apply to your submission:

The ascertainment of exposure and identification of reference group are described in the Methods (pages 10-12).

**Additional data files (“source data”)**

- We encourage you to upload relevant additional data files, such as numerical data that are represented as a graph in a figure, or as a summary table
- Where provided, these should be in the most useful format, and they can be uploaded as “Source data” files linked to a main figure or table
- Include model definition files including the full list of parameters used
- Include code used for data analysis (e.g., R, MatLab)
- Avoid stating that data files are “available upon request”

Please indicate the figures or tables for which source data files have been provided:

Numerical data used for graphs have been presented as a table below the graph (for examples, Figures 1 and 2).

The model definition, including covariates, has been thoroughly described in Methods (pages 10-11).

We have uploaded R script used for primary analyses.
